# Norisoboldine Induces Endothelium-Dependent Vasorelaxation and Attenuates Hypertension by Modulating Ca^2+^-eNOS Signaling, Oxidative Stress, and Inflammation

**DOI:** 10.3390/antiox15010131

**Published:** 2026-01-20

**Authors:** Jiaze Li, Shurui Wang, Enyi Jin, Ziyi Zhao, Jinyue Liang, Yun Jung Lee, Lihua Cao

**Affiliations:** 1 Key Laboratory of Natural Medicines of the Changbai Mountain, Ministry of Education, Medical Pharmacy, Yanbian University, Yanji 133002, China; 2023010938@ybu.edu.cn (J.L.); 17289031163@163.com (E.J.); 2024010476@ybu.edu.cn (Z.Z.); 2025010591@ybu.edu.cn (J.L.); 2 Clinical Medicine, College of Medicine, Yanbian University, Yanji 133002, China; 18607255615@163.com; 3 Department of Oriental Pharmacy, College of Pharmacy, Wonkwang-Oriental Medicines Research Institute, Wonkwang University, Iksan 54538, Republic of Korea

**Keywords:** norisoboldine, vasodilation, hypertension, oxidative stress, inflammation

## Abstract

Vascular function is a direct factor affecting blood pressure, and it is a primary strategy for clinically controlling hypertension by regulating the constriction/relaxation of blood vessels. This study evaluates the vasodilatory and anti-hypertensive effects of norisoboldine (NOR), an isoquinoline alkaloid in Ayurvedic medicine. The rat thoracic aorta was isolated to investigate the vasodilatory effect, and L-NAME-induced hypertensive rats were established, respectively. In the isolated vascular ring, removal of the endothelium resulted in a significant decrease in the vasodilatory effect. Pretreatment with L-NAME, ODQ, KT5823, WT, Tri, Dilt, calcium-free solution, TG, Gd^3+^, 2-APB, Indo, 4-AP, Gli, and BaCl_2_ inhibited the vasodilatory effect of NOR. In vascular endothelial cells, NOR promoted eNOS phosphorylation and inhibited TNF-α-induced expression of ICAM-1 and VCAM-1. SBP and DBP were significantly decreased after administration of different doses of NOR in the femoral vein of rats. In addition, NOR significantly reduced the blood pressure of L-NAME-induced hypertensive rats, up-regulated the serum levels of NO, cGMP, and CAT, and down-regulated MDA, IL-6, and TNF-α in hypertensive rats. NOR administration improved pathological changes in the thoracic aorta by regulating the arrangement of thoracic aortic smooth muscle cells, decreasing the thickness of the thoracic aortic wall, and reducing the degree of collagen deposition and fibrosis. In conclusion, the vasodilatory mechanisms of NOR were related to the Ca^2+^-eNOS signaling pathway, including the PGI_2_ and various K^+^/Ca^2+^ channels, the inositol triphosphate receptor (IP_3_R) calcium release, and the α-adrenergic receptor pathway. The anti-hypertensive mechanism of NOR may be related to increased NO and cGMP bioavailability, inhibition of oxidative stress and inflammatory responses, and improved vascular remodeling.

## 1. Introduction

Hypertension is a significant risk factor for cardiovascular diseases, including coronary heart disease, stroke, and peripheral vascular disease. Vascular tone, defined as the contractile state of vascular smooth muscle cells in small arteries and arterioles, is a primary determinant of vascular resistance and plays a crucial role in regulating blood pressure and distributing blood flow among organs [1,2]. Under pathological conditions such as hypertension, angina pectoris, and acute coronary syndromes, dysregulation of vascular tone characterized by impaired vasodilatory capacity and enhanced vasoconstriction contributes to vascular occlusion, inadequate tissue perfusion, and an increased risk of cardiovascular events [3]. Therefore, strategies aimed at improving vascular vasodilatory function have emerged as important approaches for preventing and treating cardiovascular diseases.

Vascular tone is tightly regulated through coordinated interactions between the vascular endothelium and vascular smooth muscle. The endothelium, situated at the interface between the bloodstream and vascular smooth muscle, plays a crucial role in regulating vascular tone [4]. The functional state of vascular smooth muscle cells is modulated by complex interactions among neurotransmitters, circulating hormones, and endothelium-derived vasodilatory and vasoconstrictive factors [5,6]. Oxidative stress and low-grade inflammation are recognized as key mechanisms underlying vascular aging, endothelial dysfunction, and the initiation and progression of cardiovascular diseases, including hypertension [7]. Excessive oxidative stress disrupts antioxidant defense systems and promotes the overproduction of reactive oxygen species, resulting in reduced nitric oxide (NO) bioavailability through NO scavenging and endothelial nitric oxide synthase (eNOS) uncoupling [8]. As a consequence, endothelial dysfunction impairs endothelium-dependent vasodilation, increases vascular resistance, and contributes to the development of hypertension [9].

Chronic low-grade vascular inflammation is also a critical contributor to the initiation and maintenance of hypertension [10,11]. This process is driven by the infiltration and accumulation of immune cells in target tissues, such as the kidneys and arteries, and the subsequent release of pro-inflammatory cytokines that promote vascular and renal dysfunction, leading to persistent vascular impairment and end-organ damage [12]. Notably, oxidative stress and inflammation are closely interconnected; oxidative stress-induced molecular damage and redox imbalance enhance the production of pro-inflammatory cytokines and chemokines [13,14]. Meanwhile, inflammation further exacerbates oxidative stress through the increased generation of reactive species by activated immune cells.

Although hypertension has traditionally been attributed primarily to hemodynamic abnormalities, accumulating evidence suggests that inflammatory cytokines and oxidative stress play critical roles in its progression by directly impairing vascular and renal function [15,16]. Norisoboldine (NOR), an isoquinoline alkaloid isolated from the dried tuberous roots of *Ophiopogon* species, has been reported to exhibit a broad range of biological activities. Previous studies have demonstrated that NOR possesses anti-inflammatory, immunomodulatory, and cytoprotective effects in various pathological conditions, including arthritis, colitis, and the regulation of T cells, as well as endothelial cell migration [17]. Despite these diverse pharmacological properties, the potential therapeutic effects of NOR on hypertension remain largely unexplored. Therefore, in the present study, we conducted a series of in vivo and in vitro experiments using male Sprague–Dawley rats and vascular endothelial cells to investigate the pharmacological effects of NOR. Specifically, we evaluated its vasodilatory activity in isolated rat thoracic aortic rings, its inhibitory effects on vascular inflammation, and its antihypertensive efficacy in experimental models of hypertension.

## 2. Materials and Methods

### 2.1. Drugs

**Figure 1:**
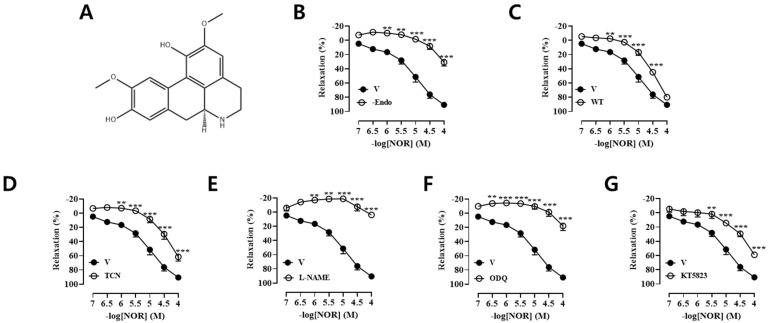
Structural formula of norisoboldine (**A**). NOR vasodilatory rates in thoracic aortic rings of endothelium-intact and endothelium-removed rats (**B**). NOR on thoracic aortic ring vasodilatory rate in wortmannin (**C**), triciribine (**D**), L-NAME (**E**), ODQ (**F**), and KT5823 (**G**) preincubated isolated rats (n = 5). ** *p* < 0.01, *** *p* < 0.001 vs. vehicle.

NOR (Figure 1A), molecular formula: C_18_H_19_NO_4_, molecular weight: 313.35, purity ≥ 98%, was purchased from Nanjing Jingzhu Biotechnology Co. (Nanjing, China). Phenylephrine (PE), acetylcholine (Ach), N^G^-nitro-L-arginine-methylester (L-NAME), glibenclamide (Gli), 1H-[1,2,4]-oxadiazolo-[4,3α]-quinoxalin-1-one (ODQ), tetraethtylamine (TEA), 4-Aminopyridine (4-AP), barium chloride (BaCl_2_), indomethacin (Indo), diltiazem (Dilt), atropine (Atro), thapsigargin (TG), propranolol (Prop), sodium nitroprusside (SNP), captopril (CPT), and KT5823 were purchase from Sigma Chemical Co. (St. Louis, MO, USA). 2-Aminoethyl diphenylborinate (2-APB), gadolinium (Gd^3+^), triciribine (TCN), and wortmannin (WT) were purchased from Biomol (Plymouth Meeting, PA, USA).

### 2.2. Experimental Animals and Cells

Male SD rats of SPF grade (body weight 250–300 g) were supplied by the Animal Centre of Yanbian University. All experiments were reviewed and approved by the Animal Care and Use Committee of Yanbian University (Protocol Number: YD20240828029; Approval Date: 28 August 2024). The entire study strictly adhered to the Regulations on the Management of Laboratory Animals and the Guidelines for the Ethical Review of Laboratory Animal Welfare (GB/T 35892-2018 [18]). Throughout the experiment, all animals were housed under controlled environmental conditions within the animal facility (12 h dark/12 h light cycle, temperature 23–25 °C, humidity 50–60%). Experimental animals were fed commercial feed, underwent overnight fasting prior to procedures, and had free access to drinking water throughout. For procedures potentially causing distress, such as surgery, appropriate anesthetics were administered strictly in accordance with the Institutional Animal Care and Use Committee’s guidelines. Following experimentation, animals were euthanised using humane methods approved by the Institutional Animal Care and Use Committee (involving administration of appropriate anesthetics followed by cervical dislocation to confirm death).

Human umbilical vein endothelial cells (HUVECs) were obtained from PromoCell (Heidelberg, Germany). HUVECs were cultured at a density of 5 × 10^5^ cells/mL in Endothelial Cell Basal Medium 2 (PromoCell, Heidelberg, Germany). Cells were incubated at 37 °C in a humidified atmosphere containing 5% CO_2_ and 95% air.

### 2.3. Preparation of Isolated Thoracic Aortic Rings

SD rats were decapitated and killed, and the thoracic cavity was opened to remove the rat thoracic aorta, which was then placed in pre-cooled Krebs’ solution at 4 °C. The fat and connective tissues were carefully removed, and the tissues were cut into ring segments of approximately 3–4 mm in length. The endothelium is removed by gently rubbing the aortic ring with a toothpick, a procedure that removes the endothelium without affecting the vessel’s smooth muscle contractility or vasorelaxation. The detached aortic rings were secured with two metal triangular rings and suspended in a bath containing 6 mL of Krebs’ solution at a constant temperature of 37 °C, which was continuously vented with 95% O_2_ and 5% CO_2_. The triangular rings were connected to a tension transducer, and changes in vascular ring tension were recorded by a BL-420S computerized biofunctional laboratory system (Chengdu Taimeng Technology Co., Ltd., Chengdu, China). Before the start of the experiment, the vascular ring should be slowly adjusted to 1 g and allowed to equilibrate for 1 h. The Krebs solution should be changed every 10 min. The endothelium was considered to be intact when the vascular ring precontracted with PE (1 × 10^−6^ M) and then treated with the addition of Ach (1 × 10^−6^ M), resulting in a vascular ring vasorelaxation of at least 80%. The endothelium was considered to be removed entirely when the vascular ring did not undergo a relaxation response.

### 2.4. P-eNOS and ICAM-1/VCAM-1 Activity

The phospho-eNOS activity was measured in HUVECs using the human P-eNOS enzyme-linked Immunosorbent assay (ELISA) kit according to the manufacturer’s protocol (ab279779, Abcam, Waltham, MA, USA). Optical density at 450 nm was used to calculate the P-eNOS activity in the samples, and results were expressed as P-eNOS/Total eNOS in (A.U). ELISA was used to determine the levels of ICAM-1 and VCAM-1 expression on the cell surface. HUVECs were seeded at a concentration of 1 × 10^5^ cells/well in 96-well plates. Briefly, HUVECs were pretreated with or without NOR for 30 min, followed by TNF-α treatment for 6 h. Following treatment, the cells were fixed with 1% paraformaldehyde and exposed to mouse anti-human ICAM-1, VCAM-1 antibody at a 1:1000 dilution in PBS containing 1% skim milk for 2 h at room temperature. The cells were washed and incubated with HRP-conjugated secondary antibody. The expression of ICAM-1 and VCAM-1 was quantified by adding a peroxidase substrate solution and measuring the absorbance of each well at 490 nm using a microplate reader (Molecular Devices, LLC, Sunnyvale, CA, USA).

### 2.5. Rat Femoral Artery Blood Pressure Measurement Experiments

After SD rats were anesthetized with urethane (1.2 mg/kg) by intraperitoneal injection, the femoral artery and femoral vein were exposed. One section of the catheter was inserted into the femoral artery, and the other end was connected to the BL-420S Biofunctional Experimental System. NOR (1 mg/kg), NOR (2 mg/kg), and NOR (4 mg/kg) were administered through the femoral vein, and an equal volume of saline containing 1% DMSO was used as a blank control. The changes in SBP and DBP before and after administration were observed and recorded.

### 2.6. L-NAME-Induced Hypertension Model

Forty-eight SD rats were randomly divided into a blank control group, an L-NAME-induced model group, a NOR low-dose group (3.75 mg/kg), a NOR medium-dose group (7.5 mg/kg), a NOR high-dose group (15 mg/kg), and a CPT group (10 mg/kg). The model group was given L-NAME (40 mg/kg/day) by gavage for 5 weeks according to the body weight of the rats, and the blank control group was given saline. The caudal arterial pressure of the rats was measured weekly with a non-invasive blood pressure meter (Jiangsu Sionce Biotech Co., Ltd., Jiangyin, China), and the modeling was considered to be successful by SBP ≥ 160 mmHg. NOR was administered intraperitoneally for 4 weeks in the high, medium, and low dose groups, as well as the CPT group. In contrast, saline was administered to the blank control and model groups. Blood pressure was measured once a week.

### 2.7. Determination of NO and cGMP Levels

Nitric oxide (NO) was determined using a nitric oxide colorimetric test kit (Elabscience Biotechnology Co., Ltd., Wuhan, China). NO in vivo or in aqueous solution is very easy to oxidize to produce NO^2−^, and with the color developer to produce light red azo compounds, the concentration of the produced azo compounds and the concentration of NO have a linear relationship. Through the colorimetric method can be indirectly calculated the concentration of NO.

Determined using the cyclic guanosine monophosphate (cGMP) enzyme-linked immunosorbent assay kit (Elabscience Biotechnology Co., Ltd.), the cGMP antibody is encapsulated on an enzyme-labeled plate, and the rat cGMP in the samples and standards binds to the antibody on the plate during the experiment. Then, the biotinylated antibody and horseradish peroxidase-labeled affinity protein are added sequentially, and they bind to rat cGMP to form an immune complex. Finally, a TMB substrate solution is added, which appears blue when catalyzed by horseradish peroxidase and turns yellow when the termination solution is added.

### 2.8. Determination of Catalase and Malondialdehyde Levels

The determination was made using a catalase (CAT) colorimetric test kit (Elabscience Biotechnology Co., Ltd.). Catalase catalyzes the decomposition of H_2_O_2_ into oxygen and water. H_2_O_2_ can interact with ammonium molybdate to produce a yellowish complex, and the amount produced can be measured at 405 nm to calculate the CAT content.

Malondialdehyde (MDA) was determined using a colorimetric test kit (Elabscience Biotechnology Co., Ltd.). MDA in the degradation products of lipid peroxidation can react with thiobarbituric acid (TBA) at high temperatures and in acidic environments to produce the reddish-brown product 3,5,5-trimethyloxazole 2,4-dione (trimethoprim), which can be determined at a wavelength of 532 nm.

### 2.9. Determination of TNF-α and IL-6 Levels

Determination was made using the Rat Tumor Necrosis Factor-α (TNF-α) enzyme-linked immunosorbent assay kit and the Rat Interleukin-6 (IL-6) enzyme-linked immunosorbent assay kit (Elabscience Biotechnology Co., Ltd.). TNF-α Antibody and IL-6 Antibody are encapsulated on an enzyme-labeled plate. TNF-α and IL-6 in samples and standards will bind to the antibodies on the plate during the experiment. Then biotinylated antibodies and horseradish peroxidase-labeled affinities are added sequentially. They will bind to TNF-α and IL-6, forming immune complexes. Finally, TMB is added, which appears blue in color, catalyzed by horseradish peroxidase, and turns yellow with the addition of termination solution.

### 2.10. Statistical Methods

Graphpad Prism 9.5.1 software was used for data processing and graphing, and data were expressed as mean ± standard error of mean (mean ± SEM). A *t*-test or one-way ANOVA test was used to compare multiple groups of data. *p* < 0.05 was statistically significant.

## 3. Results

### 3.1. Vasodilatory Effect of NOR on PE-Precontracted Endothelium Intact and Endothelium Removed from Vascular Rings

As shown in Figure 1B, the maximal vasodilatory effect of NOR on endothelium-intact vascular rings is E_max_ = 92.58 ± 1.53%, and the maximal vasodilatory effect of half effective concentration EC_50_ = 19.26 ± 1.43 μM on endothelium-removed vascular rings is E_max_ = 30.22 ± 0.92%. It was demonstrated that NOR could induce vasorelaxation in PE preconstricted vascular rings via both endothelium-dependent and non-endothelium-dependent pathways (*p* < 0.001).

### 3.2. Vasodilatory Effect of NOR on WT, TCN, L-NAME, ODQ, and KT5823 Pre-Incubated Vascular Rings

To explore whether the PI3K/AKT/eNOS/NO/cGMP/PKG signaling pathway is involved in NOR-induced vasodilation, preincubation was performed using the corresponding blockers such as PI3K inhibitor WT (1 × 10^−6^ M), AKT inhibitor TCN (5 × 10^−7^ M), NO synthase inhibitor L-NAME (1 × 10^−4^ M), the soluble guanylate cyclase inhibitor ODQ (1 × 10^−5^ M), the PKG inhibitor KT5823 (1 × 10^−7^ M), respectively (Figure 1C–G). The results showed that all of them significantly inhibited the NOR-induced vasodilatory effect (*p* < 0.001), suggesting that NOR can induce vasorelaxation through the PI3K/AKT/eNOS/NO/cGMP/PKG signaling pathway in blood vessels.

### 3.3. Vasodilatory Effect of NOR on Calcium-Free Krebs Solution and Dilt Pre-Incubated Vascular Rings

**Figure 2:**
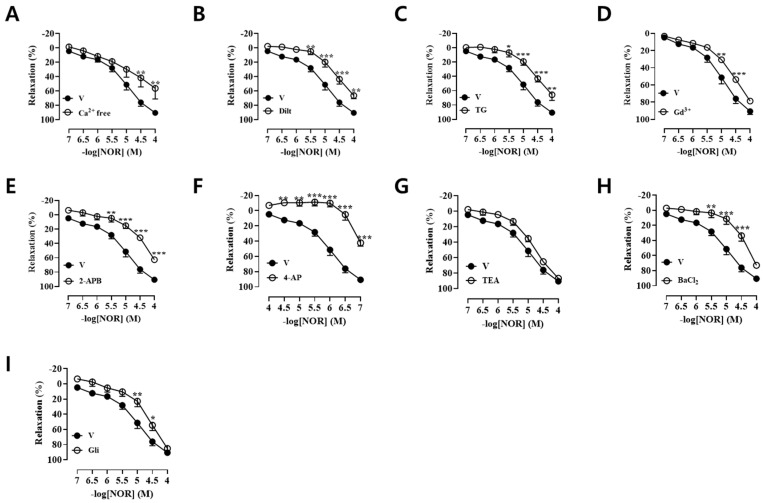
NOR vasodilatory rate of thoracic aortic rings in rats pretreated with calcium-free Krebs solution (**A**). NOR on thoracic aortic ring vasodilatory rate in diltiazem (**B**), thapsigargin (**C**), Gd^3+^ (**D**), 2-APB (**E**), 4-AP (**F**), TEA (**G**), BaCl_2_ (**H**), and glibenclamide (**I**) preincubated isolated rats (n = 5). * *p* < 0.05, ** *p* < 0.01, *** *p* < 0.001 vs. Vehicle.

To clarify whether extracellular Ca^2+^ is involved in NOR-induced vasodilation, endothelial-intact vascular rings were pretreated with calcium-free Krebs solution and the L-type calcium channel blocker Dilt (1 × 10^−5^ M), respectively. As shown in Figure 2A,B, both calcium-free Krebs solution and Dilt significantly inhibited the NOR-induced vasodilatory effect (*p* < 0.01, *p* < 0.001). It suggests that extracellular influx of Ca^2+^ is associated with NOR-induced vasodilation.

### 3.4. Vasodilatory Effect of NOR on TG, Gd^3+^, and 2-APB Preincubated Vascular Rings

To clarify the Ca^2+^-dependent vasodilatory effect of NOR, endothelial-intact vascular rings were preincubated with the sarcoplasmic reticulum calcium pump inhibitor TG (1 × 10^−7^ M), the calcium-sensitive receptor agonist Gd^3+^ (1 × 10^−5^ M), and the inositol triphosphate receptor inhibitor 2-APB (7.5 × 10^−5^ M), respectively (Figure 2C–E). The results showed that TG and 2-APB significantly inhibited the NOR-induced vasodilatory effect (*p* < 0.001). Gd^3+^ partially inhibited the NOR-induced vasodilatory effect (*p* < 0.001). It suggests that NOR can vasodilate via the Ca^2+^-eNOS signaling pathway.

### 3.5. Vasodilatory Effect of NOR on 4-AP, TEA, BaCl_2_, and Gli Pre-Incubated Vascular Rings

To investigate whether potassium channels were involved in NOR-induced vasodilation, endothelial-intact vascular rings were preincubated with four potassium channel inhibitors, respectively. The voltage-sensitive K^+^ channel (K_V_) inhibitor 4-AP (1 × 10^−3^ M) significantly inhibited the NOR-induced vasodilatory effect (*p* < 0.001) (Figure 2F). Non-selective Ca^2+^-activated K^+^ channel (K_Ca_) inhibitor TEA (1 × 10^−3^ M) did not inhibit the NOR-induced vasodilatory effect (Figure 2G). The inwardly rectifying K^+^ channel (K_IR_) inhibitor BaCl_2_ (1 × 10^−5^ M) and the ATP-sensitive K^+^ channel (K_ATP_) inhibitor Gli (1 × 10^−5^ M) partially inhibited the NOR-induced vasodilatory effect (*p* < 0.01, *p* < 0.001) (Figure 2H,I). K_V_, K_IR_, and K_ATP_ were involved in the NOR-induced vasodilatory effect, and K_Ca_ was not involved in the NOR-induced vasodilatory effect.

### 3.6. Effects of Voltage-Dependent Calcium Channels (VDCC) and Receptor-Operated Calcium Channels (ROCC) on NOR Vasodilation

**Figure 3:**
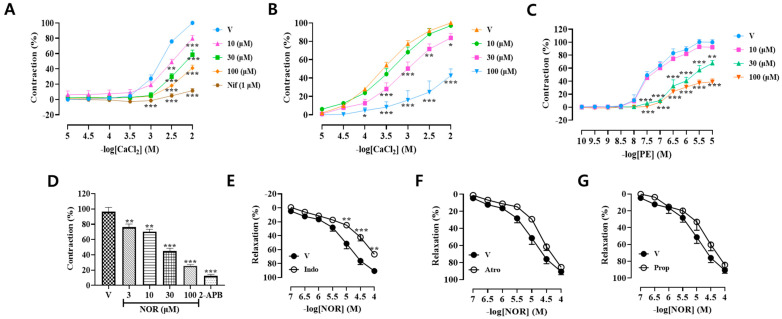
Effect of NOR in calcium-free Krebs solution on CaCl_2_-induced contraction of rat thoracic aortic rings induced by KCl (**A**). Effect of NOR in calcium-free Krebs solution on CaCl2-induced contraction of rat thoracic aortic rings induced after PE pre-contraction (**B**). The effect of NOR on sustained contraction triggered by a series of concentrations of PE (**C**). Effect of NOR in calcium-free Krebs solution on PE-induced contraction of rat thoracic aortic rings (**D**). NOR on thoracic aortic ring vasodilatory rate in indomethacin (**E**), atropine (**F**), and propranolol (**G**) preincubated isolated rats (n = 5). * *p* < 0.05, ** *p* < 0.01, *** *p* < 0.001 vs. vehicle.

High concentration of KCl promotes Ca^2+^ inward flow through VDCC. As seen in Figure 3A, NOR (10, 30, 100 μM) and Nifedipine (positive control, Nif) inhibited vasoconstriction induced by extrinsic Ca^2+^ endocytosis with significant differences (*p* < 0.001). These results suggest that NOR-induced vasodilation is associated with VDCC.

PE promotes Ca^2+^ inward flow through ROCC. NOR (30, 100 μM) was significantly different from the blank control group without KCl (*p* < 0.001) and inhibited the vasoconstriction induced by extracellular Ca^2+^ endocytosis (Figure 3B). The experimental results indicated that vasodilation induced by high concentrations of NOR was associated with ROCC. NOR (30 μM and 100 μM) had a significant difference compared with the blank control group (*p* < 0.001) and could inhibit the PE-induced concentration effect contraction (Figure 3C). The experimental results indicated that the NOR-induced vasodilatory effect was related to α-adrenergic receptors.

### 3.7. Effect of the Sarcoplasmic Reticulum IP3 Receptor (IP3R) Calcium Release Pathway on NOR Vasodilation

In a calcium-free Krebs solution, PE promoted the release of Ca^2+^ from endoplasmic reticulum stores, causing endothelial removal of vascular ring contraction via the IP_3_R calcium release pathway. As seen in Figure 3D, both NOR and 2-APB (100 μM) were significantly different (*p* < 0.001) compared with the blank control group, which suppressed vasoconstriction induced by endocannabinoid Ca^2+^ release. The results suggest that NOR-induced vasodilation is associated with the sarcoplasmic reticulum IP_3_ receptor (IP_3_R) calcium release pathway.

### 3.8. Vasodilatory Effect of NOR on Indo, Atro, and Prop Preincubated Vascular Rings

To explore whether cyclooxygenase products, muscarinic receptors, and β-adrenergic receptors are involved in NOR-induced vasodilation, endothelial-intact vascular rings were preincubated with the corresponding inhibitors. As shown in Figure 3E–G, the cyclooxygenase inhibitor Indo (1 × 10^−5^ M) inhibited the NOR-induced vasodilatory effect (*p* < 0.001), and neither the muscarinic receptor inhibitor Atro (1 × 10^−6^ M) nor the nonselective β-adrenergic receptor inhibitor Prop (1 × 10^−6^ M) inhibited the NOR-induced vasodilatory effect. The experimental results indicated that the NOR-induced vasodilatory effect was related to the prostacyclin signaling pathway, but not to muscarinic receptors and β-adrenergic receptors.

### 3.9. Inhibitory Effect of NOR on Vascular Inflammation

**Figure 4:**
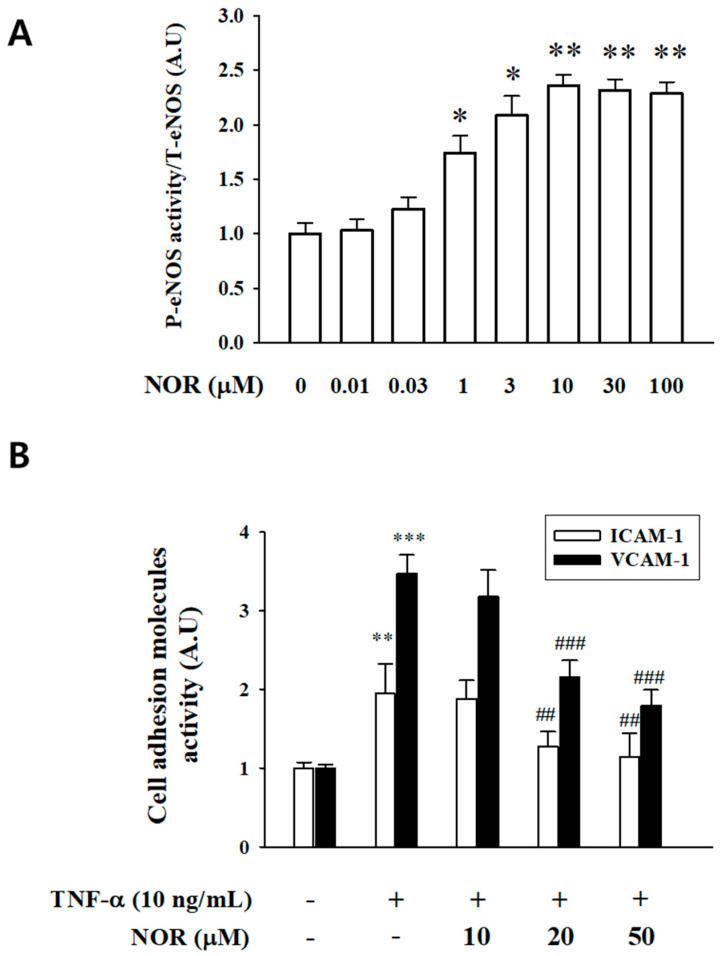
Effect of different concentrations of NOR on eNOS phosphorylation in HUVEC (**A**). Effect of NOR on ICAM-1, VCAM-1 expression (**B**) (n = 3). * *p* < 0.05, ** *p* < 0.01, *** *p* < 0.001 vs. vehicle; **^##^** *p* < 0.01, **^###^** *p* < 0.001 vs. TNF-α.

ELISA was used to detect the effect of NOR on the phosphorylation of eNOS proteins in vascular endothelial cells. In HUVEC, as shown in Figure 4A, the ratio of p-eNOS/eNOS was significantly increased after NOR treatment, indicating that NOR promotes the activation of the PI3K/AKT signaling pathway. Activity of ICAM-1 and VCAM-1 proteins was significantly up-regulated after administration of 5 ng/mL of TNF-α. However, NOR treatment significantly down-regulated the activity of ICAM-1 and VCAM-1 proteins, suggesting that NOR has some anti-inflammatory efficacy (Figure 4B).

### 3.10. Effect of NOR on Blood Pressure in Normal Rats

**Figure 5:**
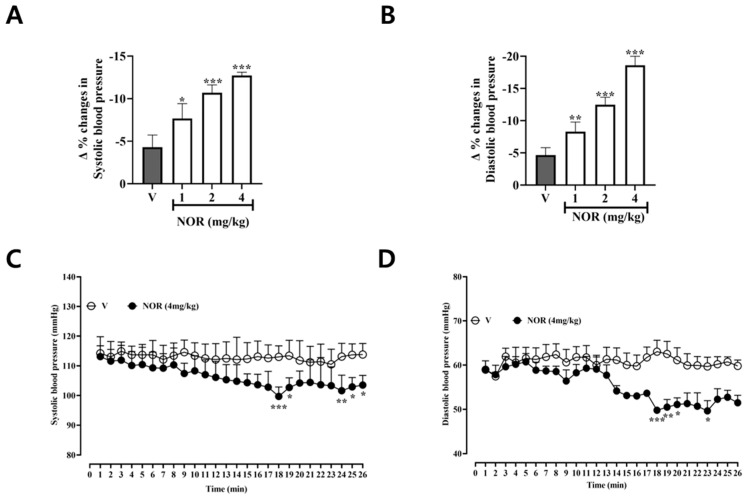
Effect of different concentrations of NOR on SBP (**A**) and DBP (**B**) in normal rats. SBP (**C**) and DBP (**D**) over time in normal rats at a NOR concentration of 4 mg/kg (n = 5). * *p* < 0.05, ** *p* < 0.01, *** *p* < 0.001 vs. vehicle.

As seen in Figure 5A,B, systolic and vasodilatory blood pressure decreased with increasing concentration of NOR in rats compared to the blank control group. After NOR administration, systolic blood pressure in rats decreased from 110.1 ± 2.13 mmHg to 99.68 ± 1.43 mmHg, and vasodilatory blood pressure in rats decreased from 60.17 ± 1.74 mmHg to 49.77 ± 0.79 mmHg, suggesting that NOR could reduce normotensive blood pressure in rats (Figure 5C,D).

### 3.11. Effect of NOR on Blood Pressure in L-NAME-Induced Hypertensive Rats

**Figure 6:**
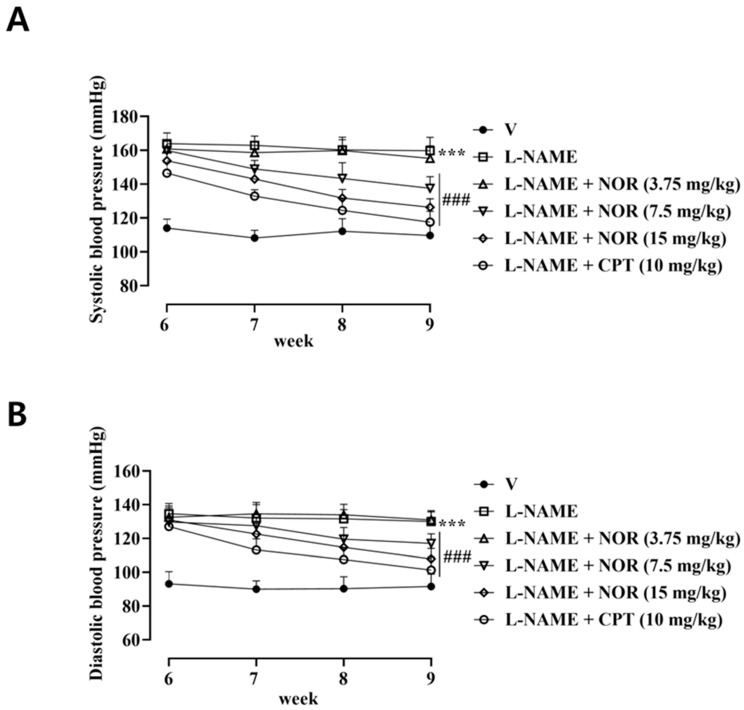
Effect of NOR on SBP (**A**) and DBP (**B**) in L-NAME-induced hypertensive rats (n = 8). *** *p* < 0.001 vs. vehicle; ^###^ *p* < 0.001 vs. model.

Hypertensive rats were treated with the positive control drug Captopril (CPT) and different doses of NOR by intraperitoneal injection. The results are shown in Figure 6. After 1 week of drug administration, SBP and DBP decreased significantly in the CPT group. After 2 weeks of drug administration, SBP and DBP decreased significantly in the NOR high-dose group. After 3 weeks of drug administration, SBP and DBP decreased significantly in the middle dose of NOR. SBP and DBP decreased to the lowest in the CPT, NOR high-dose, and NOR medium-dose groups after 4 weeks of drug administration, and SBP and DBP did not decrease significantly in hypertensive rats in the NOR low-dose group. This indicates that NOR possesses the efficacy of treating hypertension.

### 3.12. Effect of NOR on NO and cGMP in the Serum of Hypertensive Rats

**Figure 7:**
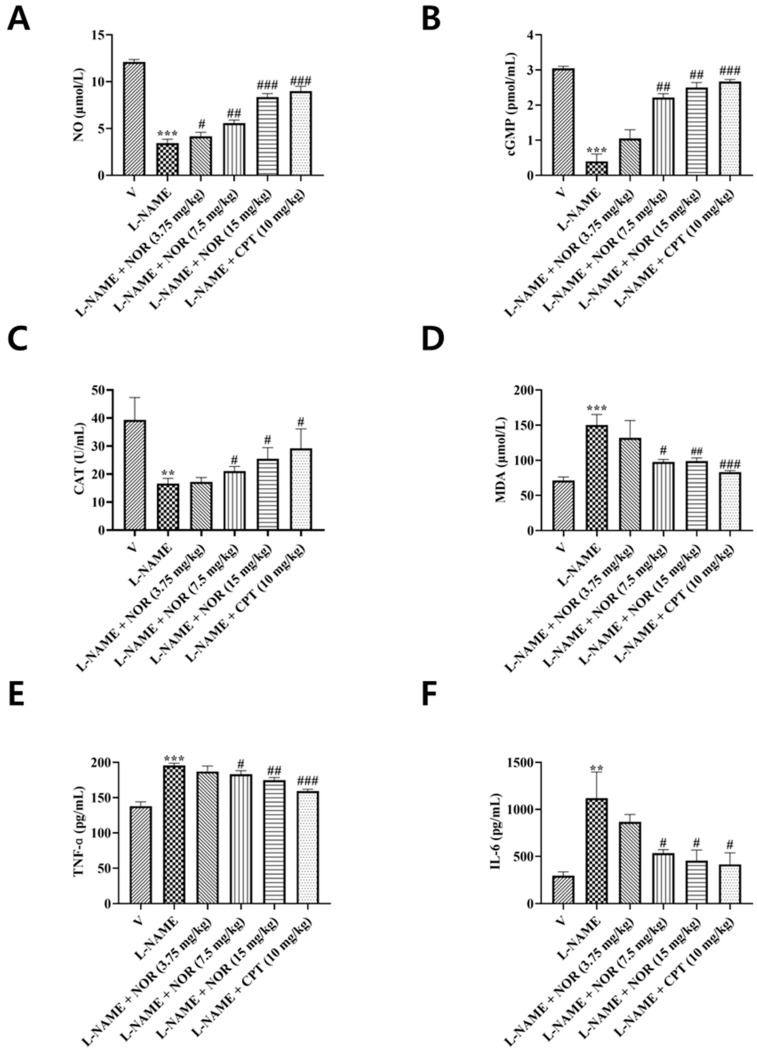
Effect of NOR on serum NO (**A**) and cGMP (**B**) in hypertensive rats. Effect of NOR on serum Catalase (**C**) and Malondialdehyde (**D**) in hypertensive rats. Effect of NOR on serum TNF-α (**E**) and IL-6 (**F**) in hypertensive rats (n = 5). ** *p* < 0.01, *** *p* < 0.001 vs. vehicle; ^#^ *p* < 0.05, ^##^ *p* < 0.01, ^###^ *p* < 0.001 vs. model.

Changes in the bioavailability of NO and cGMP can be used to assess the efficacy of drugs in hypertension. In the present study, the serum levels of NO and cGMP were measured by using the Griess method and the ELISA method in all rat groups. Compared with the control group, the serum levels of NO and cGMP were significantly reduced in the model group of rats. After drug treatment, the serum levels of NO and cGMP were significantly increased in rats in the CPT group and in the NOR group at different doses (Figure 7A,B), suggesting that NOR could exert an anti-hypertensive effect by upregulating NO and cGMP levels.

### 3.13. Effect of NOR on Oxidative Stress in the Serum of Hypertensive Rats

CAT and MDA are two classical markers of oxidative balance regulation. In order to investigate the effect of NOR on the level of oxidative stress in vivo in hypertensive rats, the present study was carried out to detect the serum levels of CAT and MDA in each group of rats by the colorimetric method. Compared with the control group, the serum content of CAT was significantly decreased and MDA content was significantly increased in the model group rats. After drug treatment, the serum content of CAT significantly increased (Figure 7C), and MDA content significantly decreased (Figure 7D) in rats in the CPT group and different doses of the NOR group, indicating that NOR has some antioxidant effects.

### 3.14. Effect of NOR on TNF-α and IL-6 in the Serum of Hypertensive Rats

TNF-α and IL-6 are two classical pro-inflammatory cytokines. To investigate the effect of NOR on the level of inflammation in hypertensive rats, the present study employed the ELISA method to detect the levels of TNF-α and IL-6 in the serum of each rat group. Compared with the control group, the serum levels of TNF-α and IL-6 were significantly increased in the model group rats. After drug treatment, the serum levels of TNF-α and IL-6 in rats in the CPT group and the NOR group at different doses decreased significantly (Figure 7E,F), indicating that NOR has some anti-inflammatory effects.

### 3.15. Effect of NOR on Thoracic Aortic Tone in Hypertensive Rats

**Figure 8:**
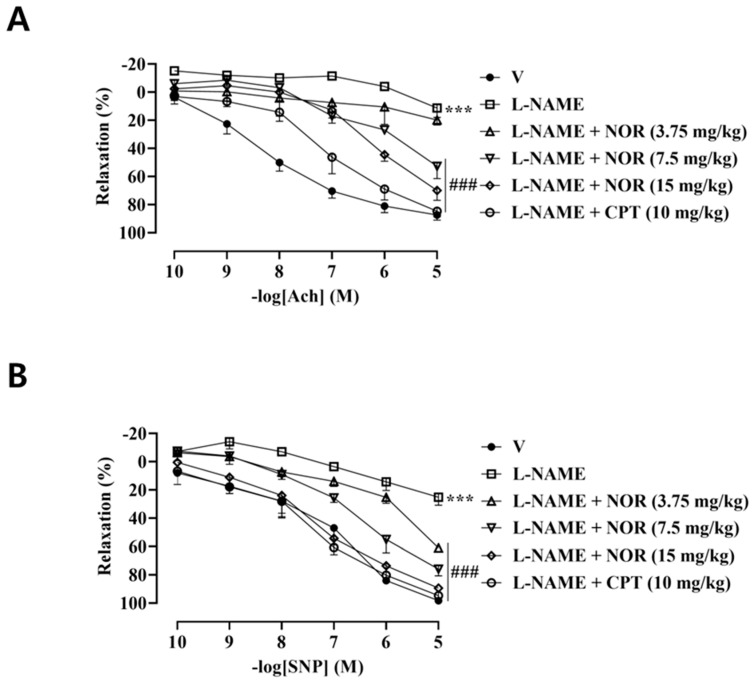
Statistical plots of the vasodilatory effect of the thoracic aorta on Ach (**A**) and SNP (**B**) in each group of rats (n = 5). *** *p* < 0.001 vs. vehicle; ^###^ *p* < 0.001 vs. model.

The vasodilatory effect of Ach and SNP on the thoracic aorta of rats in the model group was significantly reduced compared with the control group. The vasodilatory effects of Ach and SNP on the thoracic aorta of rats in the CPT group, NOR medium-dose group, and NOR high-dose group, respectively, were significantly increased after drug treatment (Figure 8). The results indicated that NOR was able to ameliorate the endothelial and smooth muscle functional impairments in the thoracic aorta of hypertensive rats.

### 3.16. Results of NOR on H&E-Stained Pathological Sections of the Thoracic Aorta of Hypertensive Rats

**Figure 9:**
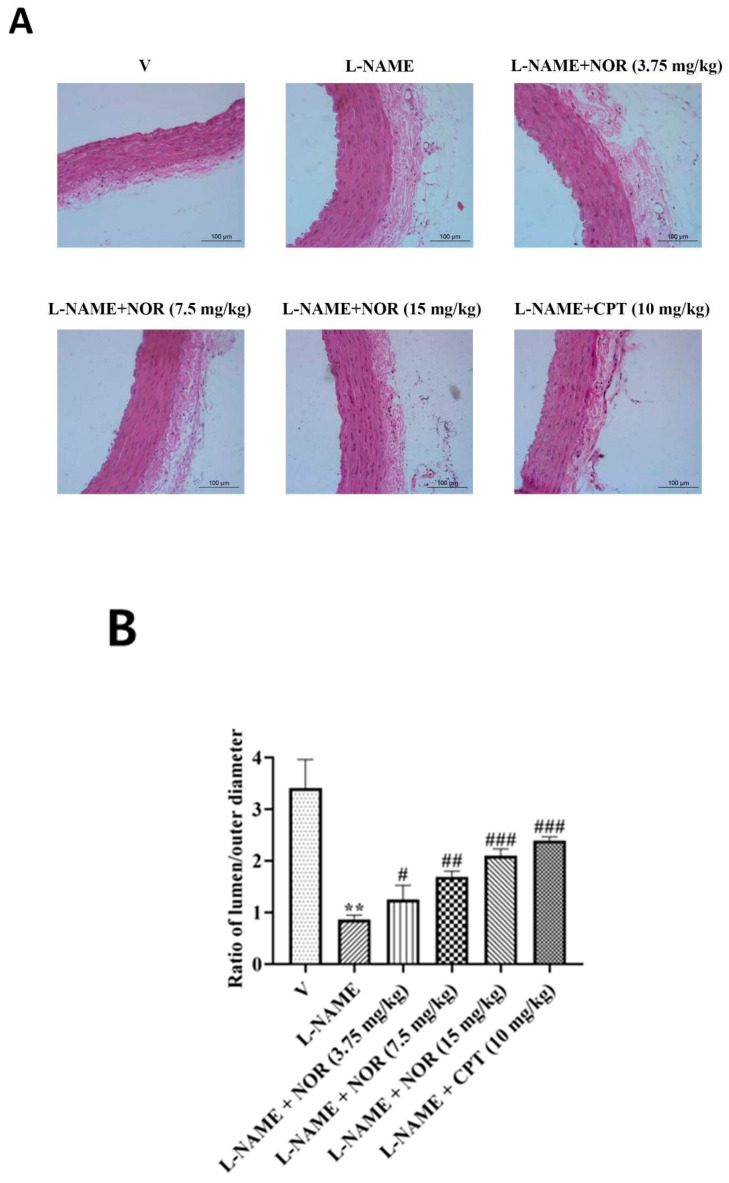

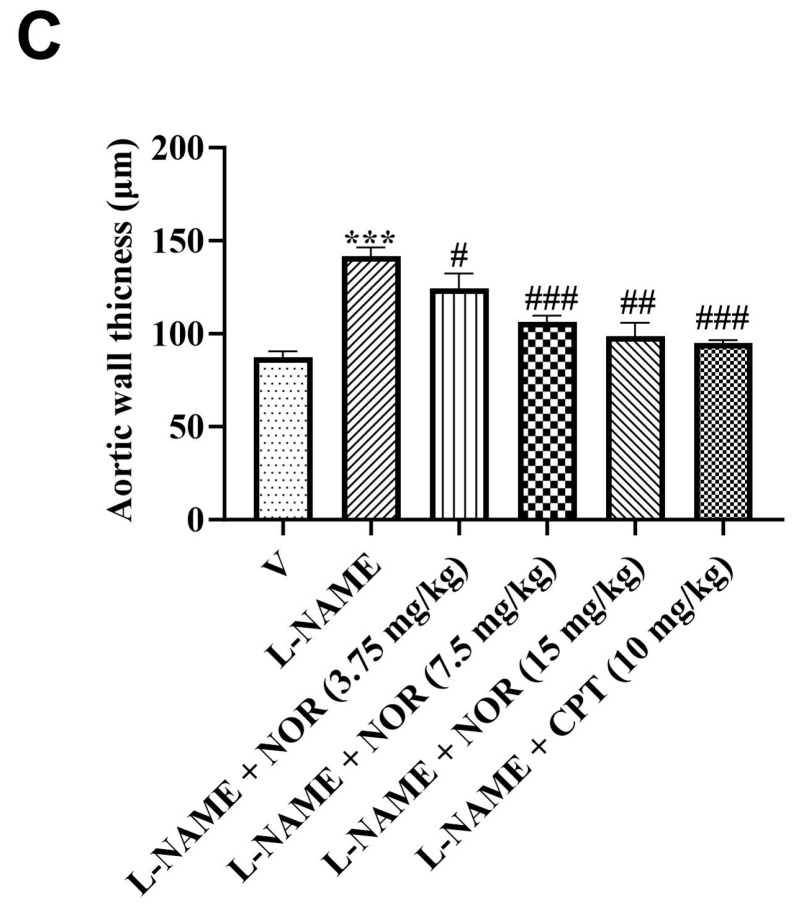
H&E staining results of rat thoracic aorta (×400) (**A**). Statistical plot of the ratio of lumen and outer diameters of the rat thoracic aortic vessel wall (**B**). Blood vessel wall thickness statistics (**C**). (n = 5). ** *p* < 0.01, *** *p* < 0.; ^#^ *p* < 0.05, **^##^** *p* < 0.01, **^###^** *p* < 0.001 vs. model.

In the model group, the smooth muscle cells of the thoracic aorta were disorganized, the inner diameter/outer diameter ratio of the vessel was significantly decreased, and the thickness of the vessel wall was significantly increased. After drug treatment, the smooth muscle cells in the thoracic aorta of rats in the CPT group and the NOR group at different doses tended to be arranged regularly, with a significant increase in the inner diameter/outer diameter ratio of the vessels and a significant reduction in the thickness of the vessel wall (Figure 9). The above results indicate that NOR improves pathological manifestations and has a significant protective effect on vascular remodeling in hypertensive rats.

### 3.17. Results of NOR on Masson-Stained Sections of the Thoracic Aorta from Hypertensive Rats

**Figure 10:**
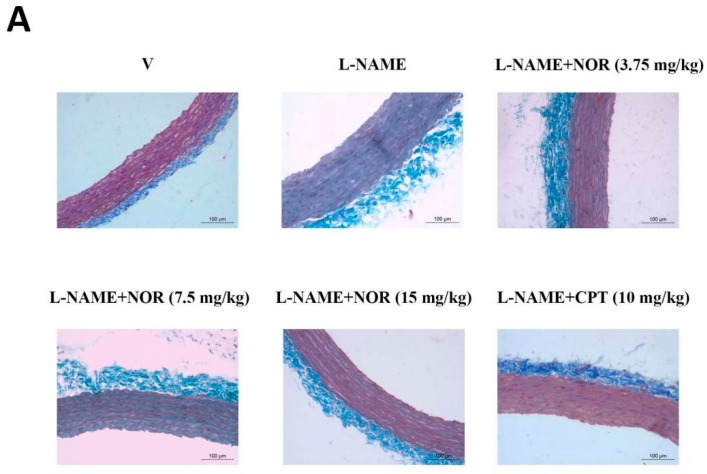

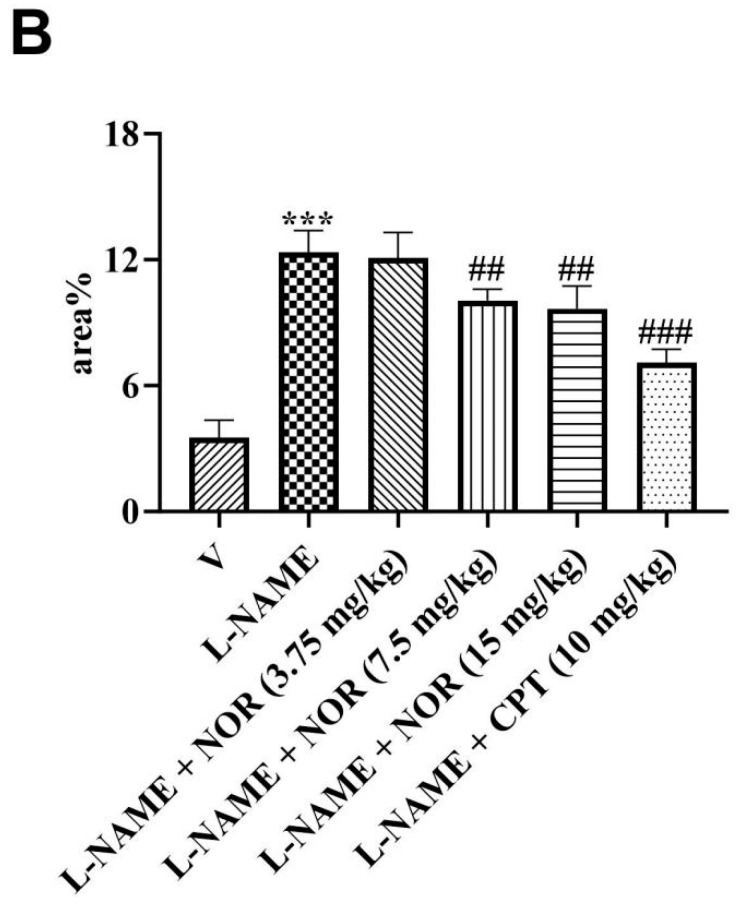
Masson staining results of rat thoracic aorta (400×) (**A**). Comparison of collagen volume fraction in rat thoracic aorta (**B**) (n = 5). *** *p* < 0.001 vs. Vehicle; ^##^ *p* < 0.01, ^###^ *p* < 0.001 vs. Model.

Masson staining showed significant collagen deposition and fibrous tissue proliferation in the vascular wall of rats in the model group compared to the control group. After drug treatment, collagen deposition and fibrosis in the vascular walls of rats in the CPT group and NOR dose groups were significantly reduced (Figure 10). These results indicated that NOR could significantly inhibit collagen deposition and fibrosis in the process of hypertension.

## 4. Discussion

Endothelial dysfunction is a key pathological feature of hypertension and reflects impaired vascular homeostasis resulting from an imbalance between endothelium-derived relaxing and contracting factors. In the present study, the vasodilatory effect of NOR is mediated by both endothelium-dependent and endothelium-independent mechanisms. Moreover, activation of the NO/sGC/cGMP/PKG pathway, together with the PGI_2_ signaling pathway, represents a primary mechanism underlying the vasodilatory effects of NOR.

NO production in endothelial cells is primarily dependent on endothelial eNOS activity, and impaired NO bioavailability is often associated with altered eNOS expression or activation [19]. eNOS activation is regulated by multiple signaling pathways, notably PI3K/AKT and Ca^2+^-dependent eNOS signaling pathways. In the vasculature, phosphorylation of eNOS represents a critical determinant of its enzymatic activity and is closely linked to activation of the PI3K/AKT signaling cascade [20,21]. In the present study, pharmacological inhibition of PI3K and AKT significantly attenuated NOR-induced vasodilation. Consistently, ELISA demonstrated that NOR markedly increased the phosphorylation levels of AKT and eNOS, as reflected by elevated p-AKT/AKT and p-eNOS/eNOS ratios. These findings suggest that NOR promotes endothelial NO production and vasodilation, at least in part, by activating the PI3K/AKT/eNOS signaling pathway.

Calcium entry into vascular endothelial cells occurs through multiple pathways, including L-type Ca^2+^ channels, store-operated calcium entry, and other calcium-permeable channels. In the present study, pretreatment with calcium-free Krebs solution, diltiazem, thapsigargin, Gd^3+^, and 2-APB significantly attenuated NOR-induced vasorelaxation, indicating that the vasodilatory effect of NOR is closely associated with activation of the Ca^2+^–eNOS–NO signaling pathway. In vascular smooth muscle cells, Ca^2+^ is a key regulator of excitation–contraction coupling, playing a central role in controlling vascular tone through dynamic changes in intracellular Ca^2+^ concentration [22,23]. Extracellular Ca^2+^ influx through VDCC and ROCC increases intracellular Ca^2+^ levels, thereby promoting vascular smooth muscle contraction [24]. The present study further demonstrated that NOR markedly inhibited CaCl_2_-induced vasoconstriction in KCl- or PE-precontracted vascular rings, as well as PE-induced vasoconstriction under calcium-free conditions. These findings suggest that NOR induces vasorelaxation not only by suppressing extracellular Ca^2+^ influx through VDCC and ROCC, but also by inhibiting intracellular Ca^2+^ release from vascular smooth muscle cells.

Vascular tone is also regulated by the arterial smooth muscle cell membrane potential through potassium K^+^ channels. Opening of K^+^ channels in vascular smooth muscle membranes promotes K^+^ efflux and triggers hyperpolarization of the membrane potential [25]. This change shuts down the VDCC, which in turn inhibits the inward flow of extracellular Ca^2+^, leading to vascular smooth muscle relaxation [26,27]. The results of the experimental study showed no significant change in the NOR-induced vasodilatory effect in endothelium-intact thoracic aortic rings after pretreatment with K_Ca_ blockers. Pretreatment with K_V_, K_ATP_, and K_IR_ inhibitors all inhibited NOR-mediated vasodilation. It is shown that K_V_, K_IR_, and K_ATP_ are involved in NOR-induced vasodilation.

Activation of muscarinic receptors stimulates the release of endothelium-derived relaxing factors, thereby inducing vasodilation. β-Adrenergic receptors, which are G protein–coupled receptors, mediate vascular smooth muscle relaxation primarily through activation of the cAMP signaling pathway. In contrast, α-adrenergic receptors are expressed on the plasma membrane of vascular smooth muscle cells and promote vasoconstriction via PE-induced activation of IP_3_R in the sarcoplasmic reticulum, leading to intracellular Ca^2+^ release [28]. In the present study, pretreatment with muscarinic receptor antagonists or β-adrenergic receptor antagonists did not significantly alter NOR-induced vasorelaxation in endothelium-intact thoracic aortic rings, indicating that muscarinic and β-adrenergic receptors are not involved in the vasodilatory effects of NOR. In contrast, higher concentrations of NOR markedly attenuated vasoconstriction induced by PE across a concentration range of 0.1 nM to 10 μM, suggesting that inhibition of α-adrenergic receptor–mediated signaling contributes to NOR-induced vasodilation.

Femoral artery cannulation is a well-established method for direct blood pressure measurement, allowing real-time monitoring of arterial pressure changes with high accuracy over short experimental periods [29,30]. Following the investigation of the vasodilatory mechanisms of NOR, the present study further evaluated its effects on systemic blood pressure in normotensive rats using femoral artery cannulation. The results demonstrated that NOR significantly reduced both SBP and DBP in a concentration-dependent manner with good reproducibility. Given that alterations in vascular tone directly influence arterial blood pressure, these findings suggest that the antihypertensive effects of NOR are closely associated with its vasodilatory activity.

In hypertensive pathologies, sustained hemodynamic stress can induce vascular endothelial cell injury, thereby triggering pronounced vascular inflammatory responses [31,32]. Vascular inflammation is driven by diverse inflammatory stimuli that promote endothelial activation, leading to increased expression of adhesion molecules, pro-inflammatory cytokines, and chemokines [33]. To determine whether NOR exerts anti-inflammatory effects on the vasculature, the expression levels of ICAM-1 and VCAM-1 were assessed by ELISA. The results demonstrated that NOR significantly downregulated TNF-α–induced ICAM-1 and VCAM-1 expression, indicating that NOR possesses pharmacological activity in suppressing vascular endothelial inflammation.

L-NAME–induced hypertension is a well-established experimental model characterized by NO deficiency and consequent endothelial dysfunction [34]. Following four weeks of treatment with captopril CPT or different doses of NOR, SBP, and DBP were significantly reduced in both the NOR low-dose and high-dose groups compared with the model group. Notably, the antihypertensive effect observed in the NOR high-dose group was comparable to that of the CPT-treated group, indicating that NOR exerts a sustained antihypertensive effect in this model.

Prolonged inhibition of NO synthesis reduces NO bioavailability, promoting oxidative stress and inflammatory responses that exacerbate hypertension and vascular dysfunction [35,36]. In the present study, NOR administration markedly increased serum levels of NO, cGMP, and catalase, while significantly reducing malondialdehyde, TNF-α, and IL-6 levels in hypertensive rats. In addition, NOR treatment enhanced the vasodilatory capacity of the thoracic aorta and markedly improved vascular structural abnormalities induced by hypertension. Histological analysis revealed a more regular arrangement of vascular smooth muscle cells, an increased lumen-to-wall ratio, reduced vascular wall thickness, decreased collagen deposition, and attenuated vascular fibrosis. These findings suggest that the antihypertensive effects of NOR are mediated through restoration of NO–cGMP signaling, suppression of oxidative stress and inflammatory responses, improvement of pathological vascular remodeling, and recovery of endothelial and vascular smooth muscle vasodilatory function.

Current research has systematically elucidated the vasodilatory and antihypertensive effects of norisoprodine (NOR) alongside its potential mechanisms. Nevertheless, certain limitations and constraints remain. Firstly, studies predominantly rely on in vitro experiments using rat thoracic aortic rings and L-NAME-induced hypertensive models. These findings face limitations in generalizability, as vascular reactivity may exhibit significant variations across different species, vascular beds, and pathophysiological states. Secondly, within methodological scope, although multiple techniques, including vasotensiometry, serum biochemical assays, and histopathological staining, were employed, these experiments predominantly consisted of short-term observations. Moreover, the molecular mechanisms underpinning the drug’s effects require further validation through more in-depth cellular signaling pathway studies (such as examining phosphorylation levels of additional key proteins and conducting gene expression analyses). Moreover, the HUVEC inflammation model reflects anti-inflammatory effects solely at the endothelial cell level, failing to replicate the complex inflammatory microenvironment in vivo fully. Finally, regarding clinical applicability, discrepancies exist between animal studies and human conditions concerning dosage, administration routes, and disease models. Furthermore, the absence of long-term toxicity, pharmacokinetic, and pharmacodynamic data necessitates further human clinical trials to assess NOR’s translational value. The outline of proposed follow-up research is as follows: (1) validating the direct binding capacity of NOR to the PI3K/AKT signaling pathway (a primary vasodilatory pathway) through in vitro cellular experiments; (2) clarifying the dependence of pharmacological effects on relevant signaling pathways via strategies such as pathway inhibitor intervention and gene knockout; (3) expanding research to examine whether inflammatory and oxidative stress-related factors participate in NOR’s therapeutic effects on hypertension. Overall, this study provides a crucial foundation for the pharmacological activity of NOR. However, extrapolation of its conclusions requires caution and should be further validated through broader experimental systems and preclinical studies.

## 5. Conclusions

The vasodilatory effect of NOR on blood vessels is associated with the NO/sGC/cGMP/PKG signaling pathway activated via the Ca^2+^-eNOS and PGI_2_ signaling pathway, K_V_, K_ATP_, K_IR_, VDCC, ROCC, the IP_3_R calcium release pathway, and the α-adrenergic receptor pathway. The vasodilatory mechanisms of NOR were related to the Ca^2+^-eNOS signaling pathway, including the PGI_2_ and various K^+^/Ca^2+^ channels, the inositol triphosphate receptor (IP_3_R) calcium release, and the α-adrenergic receptor pathway. The anti-hypertensive mechanism of NOR may be related to an increase in NO and cGMP bioavailability, inhibition of oxidative stress and inflammatory responses, and improved vascular remodeling.

## Data Availability

The data presented in this study are available on request from the corresponding author.

## References

[1] Godfraind T. (2005). Antioxidant effects and the therapeutic mode of action of calcium channel blockers in hypertension and atherosclerosis. Philos. Trans. R. Soc. Lond. B Biol. Sci..

[2] Jackson W.F. (2000). Ion channels and vascular tone. Hypertension.

[3] Owen R.S., Carpenter J.P., Baum R.A., Perloff L.J., Cope C. (1992). Magnetic resonance imaging of angiographically occult runoff vessels in peripheral arterial occlusive disease. N. Engl. J. Med..

[4] Lee S.M., Lee Y.J., Choi J.H., Kho M.C., Yoon J.J., Shin S.H., Kang D.G., Lee H.S. (2014). Gal-geun-dang-gwi-tang improves diabetic vascular complication in apolipoprotein E knockout mice fed a western diet. BMC Complement. Altern. Med..

[5] Nava E., Llorens S. (2019). The local regulation of vascular function: From an inside–outside to an outside–inside model. Front. Physiol..

[6] Lee K., Shin M.S., Ham I., Choi H.-Y. (2015). Investigation of the mechanisms of *Angelica dahurica* root extract-induced vasorelaxation in isolated rat aortic rings. BMC Complement. Altern. Med..

[7] Valenzuela P.L., Carrera-Bastos P., Gálvez B.G., Ruiz-Hurtado G., Ordovas J.M., Ruilope L.M., Lucia A. (2021). Lifestyle interventions for the prevention and treatment of hypertension. Nat. Rev. Cardiol..

[8] Carlström M., Lundberg J.O., Weitzberg E. (2018). Mechanisms underlying blood pressure reduction by dietary inorganic nitrate. Acta Physiol..

[9] Fejes R., Pilat N., Lutnik M., Weisshaar S., Weijler A.M., Krüger K., Draxler A., Bragagna L., Peake J.M., Woodman R.J. (2024). Effects of increased nitrate intake from beetroot juice on blood markers of oxidative stress and inflammation in older adults with hypertension. Free Radic. Biol. Med..

[10] Zewinger S., Reiser J., Jankowski V., Alansary D., Hahm E., Triem S., Klug M., Schunk S.J., Schmit D., Kramann R. (2020). Apolipoprotein C3 induces inflammation and organ damage by alternative inflammasome activation. Nat. Immunol..

[11] Guzik T.J., Touyz R.M. (2017). Oxidative stress, inflammation, and vascular aging in hypertension. Hypertension.

[12] Raubenheimer K., Bondonno C., Blekkenhorst L., Wagner K.-H., Peake J.M., Neubauer O. (2019). Effects of dietary nitrate on inflammation and immune function, and implications for cardiovascular health. Nutr. Rev..

[13] Olivares-Silva F., De Gregorio N., Espitia-Corredor J., Espinoza C., Vivar R., Silva D., Osorio J.M., Lavandero S., Peiró C., Sánchez-Ferrer C. (2021). Resolvin D1 attenuates angiotensin II-induced cardiac inflammation and prevents cardiac remodeling and hypertension in mice. Biochim. Biophys. Acta Mol. Basis Dis..

[14] Förstermann U., Xia N., Li H. (2017). Roles of vascular oxidative stress and nitric oxide in the pathogenesis of atherosclerosis. Circ. Res..

[15] Zhang Z., Zhao L., Zhou X., Meng X., Zhou X. (2022). Role of inflammation, immunity, and oxidative stress in hypertension: New insights and potential therapeutic targets. Front. Immunol..

[16] Yang J., Villar V.A.M., Jose P.A., Zeng C. (2021). Renal dopamine receptors and oxidative stress: Role in hypertension. Antioxid. Redox Signal..

[17] Patel D.K., Patel K. (2023). Therapeutic importance and pharmacological activities of norisoboldine in medicine for the treatment of human disorders. Drug Metab. Bioanal. Lett..

[18] (2018). Laboratory Animal—Guideline for Ethical Review of Animal Welfare.

[19] Parkington H.C., Coleman H.A., Tare M. (2004). Prostacyclin and endothelium-dependent hyperpolarization. Pharmacol. Res..

[20] Púzserová A., Kopincová J., Bernátová I. (2008). The role of endothelium and nitric oxide in the regulation of vascular tone. Cesk. Fysiol..

[21] Ohkita M., Tawa M., Kitada K., Matsumura Y. (2012). Pathophysiological roles of endothelin receptors in cardiovascular diseases. J. Pharmacol. Sci..

[22] Liang X.X., Wang R.Y., Guo Y.Z., Cheng Z., Lv D.Y., Luo M.H., He A., Luo S.-X., Xia Y. (2021). Phosphorylation of Akt at Thr308 regulates eNOS Ser1177 phosphorylation under physiological conditions. FEBS Open Bio.

[23] Su K.H., Lin S.J., Wei J., Lee K., Zhao J., Shyue S., Lee T. (2014). Essential role of transient receptor potential vanilloid 1 in simvastatin-induced activation of endothelial nitric oxide synthase and angiogenesis. Acta Physiol..

[24] Razali N., Dewa A., Asmawi M.Z., Mohamed N., Manshor N.M. (2020). Mechanisms underlying vascular relaxation induced by *Zingiber officinale* var. *rubrum* in thoracic aorta of spontaneously hypertensive rats. J. Integr. Med..

[25] Assis K.S., Araújo I.G.A., De Azevedo F., Maciel P.M.P., Calzerra N.T.M., da Silva T.A.F., Assis V.L., de Vasconcelos A.P., Santos C.A.G., Meireles B.R.L.A. (2018). Potassium channel activation is involved in cardiovascular effects induced by freeze-dried *Syzygium jambolanum* fruit juice. BioMed Res. Int..

[26] Vanhaesebroeck B., Leevers S.J., Panayotou G., Waterfield M.D. (1997). Phosphoinositide 3-kinases: A conserved family of signal transducers. Trends Biochem. Sci..

[27] Dogan M.F., Yildiz O., Arslan S.O., Ulusoy K.G. (2019). Potassium channels in vascular smooth muscle: A pathophysiological and pharmacological perspective. Fundam. Clin. Pharmacol..

[28] Tangsucharit P., Takatori S., Zamami Y., Goda M., Pakdeechote P., Kawasaki H., Takayama F. (2016). Muscarinic acetylcholine receptor M1 and M3 subtypes mediate acetylcholine-induced endothelium-independent vasodilatation in rat mesenteric arteries. J. Pharmacol. Sci..

[29] Tanaka Y., Horinouchi T., Koike K. (2005). New insights into β-adrenoceptors in smooth muscle: Distribution of receptor subtypes and molecular mechanisms triggering muscle relaxation. Clin. Exp. Pharmacol. Physiol..

[30] Jin J., Shen X., Tai Y., Li S., Liu M., Zhen C., Xuan X., Zhang X., Hu N., Zhang X. (2017). Arterial relaxation is coupled to inhibition of mitochondrial fission in arterial smooth muscle cells: Comparison of vasorelaxant effects of verapamil and phentolamine. Acta Pharm. Sin. B.

[31] Gimbrone M.A., García-Cardeña G. (2016). Endothelial cell dysfunction and the pathobiology of atherosclerosis. Circ. Res..

[32] Jing Y., Wang G., Xiao Q., Zhou Y., Wei Y., Gong Z. (2018). Antiangiogenic effects of AA-PMe on HUVECs in vitro and zebrafish in vivo. OncoTargets Ther..

[33] Pletsch-Borba L., Watzinger C., Turzanski Fortner R., Katzke V., Schwingshackl L., Sowah S.A., Hüsing A., Johnson T., Groß M.-L., Maldonado S.G. (2019). Biomarkers of vascular injury and type 2 diabetes: A prospective study, systematic review and meta-analysis. J. Clin. Med..

[34] Helle F., Iversen B.M., Chatziantoniou C. (2010). Losartan increases nitric oxide release in afferent arterioles during regression of L-NAME-induced renal damage. Am. J. Physiol. Renal Physiol..

[35] Silambarasan T., Manivannan J., Krishna Priya M., Suganya N., Chatterjee S., Raja B. (2014). Sinapic acid prevents hypertension and cardiovascular remodeling in nitric oxide-deficient rats. PLoS ONE.

[36] Bunbupha S., Prachaney P., Kukongviriyapan U., Kukongviriyapan V., Welbat J.U., Pakdeechote P. (2015). Asiatic acid alleviates cardiovascular remodeling in rats with L-NAME-induced hypertension. Clin. Exp. Pharmacol. Physiol..

